# Relationship of Medication Intake and Systemic Conditions with Periodontitis: A Retrospective Study

**DOI:** 10.3390/jpm13101480

**Published:** 2023-10-10

**Authors:** Georgios S. Chatzopoulos, Ziou Jiang, Nicholas Marka, Larry F. Wolff

**Affiliations:** 1 Division of Periodontology, Department of Developmental and Surgical Sciences, School of Dentistry, University of Minnesota, Minneapolis, MN 55455, USA; wolff001@umn.edu; 2 Department of Preventive Dentistry, Periodontology and Implant Biology, Faculty of Dentistry, School of Health Sciences, Aristotle University of Thessaloniki, 54124 Thessaloniki, Greece; 3 Biostatistical Design and Analysis Center, Clinical and Translational Science Institute, University of Minnesota, Minneapolis, MN 55414, USA; jian0717@umn.edu (Z.J.); marka007@umn.edu (N.M.)

**Keywords:** drug therapy, oral health, oral-systemic disease, periodontal disease, tooth loss

## Abstract

Objectives: To examine the potential relationship of medication intake and systemic conditions with periodontitis. Methods and Materials: A total of 1985 patient records with a diagnosis of periodontal health and stage III and IV periodontitis were included in the analysis. Demographic characteristics, the number of missing teeth, patient-reported medical conditions and medication intake as well as smoking habits were recorded. Regression models were performed to explore the outcomes. Results: Older individuals, Hispanic ethnic groups, Black and Hispanic or Latino racial groups and non-White individuals in general were significantly more frequently diagnosed with periodontitis than health. Hypertension, glaucoma, anxiety and depression were significantly associated with periodontitis, while cancer, alcohol use, kidney problems, asthma, sleep apnea and gastrointestinal disorders were associated with periodontal health. Patients who reported taking anticoagulants, statins and ACE inhibitors demonstrated 3.546 (95% CI: 1.982, 6.343), 2.771 (95% CI: 1.877, 4.09) and 4.847 (95% CI: 2.785, 8.434) times higher odds of having periodontitis, respectively. Conclusion: Within the limitations of this retrospective study that utilized the BigMouth dental data repository, there is a possible relationship between systemic medications including anticoagulants, ACE inhibitors and statins as well as systemic medical conditions including hypertension, glaucoma, anxiety and depression with periodontitis.

## 1. Introduction

Periodontal diseases are inflammatory diseases that exhibit multifactorial etiology and are initiated by microbial pathogens leading to tooth-supporting tissue destruction [1]. The development and progression of periodontal disease are a result of the interaction between pathogenic microorganisms as well as other parameters including genetics, environmental and lifestyle factors and the host-immune response [2,3]. Imbalances in the microbiota–immunity interactions which lead to the disruption of homeostasis and therefore disease onset may be affected by the systemic health condition of an individual [4].

Periodontitis development and progression may ultimately lead to tooth loss and edentulism, which may further cause masticatory dysfunction, poor nutrition, compromised speech and reduced quality of life [5]. This poses a significant socio-economic impact as well as increased healthcare costs [5]. Periodontal disease has a high prevalence globally and it is considered the sixth-most prevalent health condition, while its severe form of disease was estimated to affect 10.8% of the global population [6]. From 1990 to 2019, the global incidence of periodontitis increased by 83.4% revealing that current measures for periodontitis prevention are inadequate in controlling the disease worldwide [7]. In addition, the costs posed by periodontal disease in the United States and in 32 European countries in 2018 was estimated to be USD 154.06 billion and EUR 158.64 billion that year, respectively [8].

Over the years, a number of systemic conditions have been associated with periodontal disease. This has led to the development of a new term, “periodontal medicine”, to describe the relationship between systemic health and periodontal disease [9]. Periodontal disease has been linked to 57 systemic conditions, with the most common being diabetes, cardiovascular disease, obesity and preterm birth [10]. In addition, cancer, osteoporosis, thyroid disorders, depression, COPD, HIV, obstructive sleep apnea, pemphigus/pemphigoid and Down syndrome have been examined for their potential association with periodontal disease [11]. It is worth noting that periodontitis has been independently associated with the vast majority of chronic noncommunicable diseases of aging and premature mortality [12]. Tobacco, alcohol, medications, uncontrolled diabetes and obesity have been suggested as factors that should be taken into consideration for a personalized care model for the management of periodontal diseases [13]. In a cross-sectional pilot study from the Netherlands, periodontal disease was highly prevalent in multimorbidity patients, and significant associations were found in patients with cardiovascular diseases [14]. Systemic inflammatory conditions including hypertension, arthritis, asthma, diabetes and HIV were also found to be significantly associated with the severity of bone loss as well as diabetes, lupus and the extent of missing teeth in a retrospective study that included 4890 randomly selected patients [15]. Tobacco use has been recognized as a significant factor affecting periodontal disease onset, progression and treatment outcomes for several years [16,17]. Chronic inflammatory diseases share common causal factors including genetics and immune factors, which may explain their relationship [18]. Differences in the reported findings regarding the periodontal–systemic associations may be attributed to the inclusion of patients with a different severity and degree of systemic disease-control. Medication intake may reflect systemic disease severity and may explain their association with periodontitis. 

A number of effects on periodontal tissues have been reported in the literature as a result of medication intake. Gingival enlargement, inflammation, pigmentations, gingival bleeding and osteonecrosis have been recorded. Drug-induced gingival overgrowth or enlargement is highly prevalent in patients taking phenytoin (anticonvulsant), cyclosporine (immunosuppressant), nifedipine and amlodipine (calcium channel blockers) [19]. Minocycline has been associated with pigmentations of the oral mucosa including buccal mucosa, gingiva, palates, lips and tongues [20]. Anti-platelets including aspirin and clopidogrel may increase gingival bleeding, especially following surgical treatment [21]. Bisphosphonates may also increase the risk of osteonecrosis following invasive dental treatment, while they may also be used locally as an adjunct treatment for scaling and root planing due to their antiresorptive properties [22,23]. A retrospective case-control study was aimed at investigating the potential association between medication consumption and the severity of periodontitis. Hypoglycemic agents, calcium channel blockers, insulin and diuretics were significantly higher in the periodontitis patients, while a higher intake frequency of angiotensin-converting enzyme (ACE) inhibitors and antidepressants was also found in the periodontitis group [24].

Early diagnosis has the potential to improve quality of life, preserve the natural dentition, maintain masticatory function and ultimately reduce the social and financial burden associated with periodontitis [5]. It is critically important to recognize systemic factors of an individual such as medications and inform the dental and medical community for potential associations with oral conditions such as periodontitis. Limited information is currently available in the literature surrounding the potential differences in regard to the medication intake between periodontitis and periodontally healthy individuals. Therefore, the aim of the present investigation was to examine the potential relationship of medication intake and systemic conditions with periodontitis.

## 2. Materials and Methods

This retrospective study was reviewed by the Institutional Review Board of the University of Minnesota, and it was determined that it is not research involving human subjects, as defined by the Department of Health and Human Services as well as the United States Food and Drug Administration (STUDY00016576). It was further reviewed and approved by the BigMouth Consortium for Oral Health Research and Informatics clinical review committee. This study was conducted in agreement with the Helsinki Declaration of 1975, as most recently revised in 2013. Data from the BigMouth Dental Data Repository used for this study were deidentified, and no extraction of personally identifiable information occurred. No recruitment of any patients and no access to patient identifiers were performed. The need for consent was thus waived. 

The BigMouth Dental Data Repository was used to extract electronic health records from 2011 to 2021. Dental charts of adult patients who, seeking dental therapy, had attended the dental clinics of the universities contributing data to the BigMouth network and who accepted the protocol of the study were evaluated: Harvard University; University of Texas Health; The University of California, San Francisco; University of Colorado; Loma Linda University; University of Buffalo; The University of Iowa and The University of Minnesota. Electronic health records were entered during the patients’ visits by both dental students, residents and faculty oral healthcare providers. BigMouth is a centralized data repository that includes deidentified clinical data extracted from electronic health records at multiple institutions. Each participating institution controls the data it contributes and provides a “limited dataset” with deidentified data only. The technical team of BigMouth assesses the data quality when extracted from the participating institutions and after the load process through an automated script which compares the data received from all institutions with the previous extracts from the same institution. A system sanity checklist is also used to assess both the data quality and functionality of the BigMouth interface. 

Dental Procedure Codes and Current Procedural Terminology procedures were utilized. Patients with at least one completed treatment code D0150 (comprehensive oral evaluation), D0120 (periodic oral evaluation provided to an established patient) or D0180 (comprehensive restorative and periodontal exam) were included. Only patients who completed any of the D0150, D0120 and D0180 treatment codes were included to ensure that included patients had comprehensive examination at any of the participating sites. Thus, patients who only had urgent treatment or limited oral examination were not included in the records. Patients’ records identified in the BigMouth Dental Data Repository with a periodontal diagnosis based on the 2017 World Workshop on the Classification of Periodontal and Peri-Implant Diseases and Conditions were evaluated [25,26]. Initially, periodontitis patients were identified based on the following procedure codes: “D4210” or “D4211” or D4240” or “D4241” or “D4245” or “D4260” or “D4261” or “D4263” or “D4266” or “D4274” or “D4341” or “D4342” or “D4910”, which are used for the non-surgical and surgical treatment of periodontal disease. From the total population who had any of the above-mentioned treatment codes, individuals with stage III and stage IV periodontitis were identified by the use of the Dental Diagnostic System (DDS) (formerly known as EZCodes, which contains dental diagnostic terms), and they were solely included in the periodontitis group. 

Individuals were categorized into periodontal health and periodontitis based on the periodontal diagnosis included in the electronic health records of the eligible dental records as follows:

- Periodontal health: Gingival health is defined as <10% bleeding sites with probing depths ≤ 3 mm and no radiographic evidence of bone loss [25].
- Periodontitis: Individuals with stage III and IV were included, who exhibit interdental clinical attachment loss of ≥5 mm and radiographic bone loss extending to the middle third of the root and beyond.

Data were extracted and validated from the electronic health records by independent data analysts at The University of Texas Health Science Center at Houston. Extracted data included:

- Demographic characteristics: age (at baseline); ethnicity (Hispanic, non-Hispanic, other); race (Asian, African American, Pacific islander, American/Indian/Alaskan native, White, Hispanic or Latino, other); sex (male, female).
- Number of missing teeth (at baseline).
- Patient-reported medical conditions included the following: alcohol consumption, blood and hematological disorders, cancer, angina, congenital heart disease, coronary heart disease, history of endocarditis, heart attack, high blood pressure, implant defibrillator, rheumatic fever, smoking, cocaine use, marijuana use, adrenal gland disorder, diabetes, thyroid disorders, glaucoma, HIV, sexually transmitted disease, dialysis, kidney disease, renal failure or insufficiency, arthritis, lupus, osteoporosis, dementia, anxiety, depression, Parkinson disease, multiple sclerosis, seizure or epilepsy, stroke, organ transplantation, asthma, chronic bronchitis or emphysema, sleep apnea, Crohn’s disease, gastrointestinal disorder and hepatitis. Self-reported systemic diseases are based on a patient questionnaire that is completed at the initial visit and updated every 6–12 months.
- Self-reported smoking habits (yes/no), as reported in the medical history form.
- Patient-reported medication intake including: ACE inhibitors (Benazepril, Enalapril, Lisinopril, Quinapril, Ramipril); Antidepressants (fluoxetine, paroxetine, sertraline, citalopram, duloxetine, venlafaxine, desvenlafaxine, trazodone, mirtazapine, bupropion, nortriptyline, doxepin, quetiapine, aripiprazole, lithium, Amitryptiline); Anti-coagulants (Apixaban, Plavix, Xarelto, Brilinta, Cilostazol, Aspirin, Clopidogrel, Warfarin); Statins (Atorvastatin, Lovastatin, Pravastatin, Rosuvastatin, Simvastatin); Biphosphonates (Zoledronic acid, ibandronate, alendronate, risedronate) and Proton Pump Inhibitors (Omeprazole, Lansoprazole, Dexilant, Esomeprazole, Pantoprazole).

### Statistical Analysis

Patients’ demographic and clinical characteristics were summarized as means and standard deviations for continuous variables and as frequencies and percentages for categorical variables. Normality was assessed using density plots. Between healthy and periodontal disease patients, two sample *t*-tests and Chi-square tests were used for the comparison of continuous and categorical characteristics, respectively. Univariate associations between systemic medical conditions, medication intake and periodontal disease status were assessed using a logistic regression model, with the results presented as Odds Ratios (OR) and their corresponding 95% confidence intervals (CI). Multivariable regression models were explored for the above outcomes, adjusting for the effects of age, sex and missing teeth at baseline. For all multivariable regression models, *p*-values were corrected for multiple comparisons using the Hochberg method. All analyses were based on complete cases and were conducted at the 0.05 significance level using the R environment (R Foundation for Statistical Computing, Vienna, Austria (Version 4.2.1).

## 3. Results

The demographic characteristics of the included population are shown in Table 1. A total of 1985 patient records with a diagnosis of periodontal health and stage III and IV periodontitis according to the 2017 World Workshop on the Classification of Periodontal and Peri-Implant Diseases and Conditions were included in the analysis. Out of these records, 1099 patients were diagnosed with periodontitis (stage III and IV) and 886 with periodontal health. Significant differences were identified between the two groups with respect to age (*p* < 0.001), ethnicity (*p* < 0.001), race (*p* < 0.001) as well as the number of missing teeth (*p* < 0.001). Older individuals, Hispanic ethnic groups, Black and Hispanic or Latino racial groups and non-White individuals in general were significantly more frequently diagnosed with periodontitis than health. Periodontitis patients showed a significantly higher number of missing teeth than periodontally healthy individuals (*p* < 0.001).

The examined self-reported systemic medical conditions as well as alcohol use and smoking habits compared between periodontal health and periodontitis individuals are presented in Table 2. Forty-seven medical conditions as well as alcohol use, smoking habits including cigarettes, marijuana use, methamphetamine use and cocaine use were included in the analysis. Coronary heart disease, hypertension, implant defibrillator, marijuana use, diabetes, glaucoma, sexually transmitted diseases, arthritis, osteoporosis, depression, hepatitis and rheumatic fever were significantly (*p* < 0.005) more frequently reported by periodontitis patients. Cancer, alcohol use, asthma, sleep apnea, gastrointestinal disorders, anemia, bleeding disorders, cardiovascular diseases, anorexia, bulimia, bronchitis, respiratory or lung problems, sinusitis, tuberculosis and cirrhosis or chronic hepatitis were significantly (*p* < 0.005) more prevalent in the periodontally healthy patient group. 

The multivariable regression analysis for the association between systemic medical conditions, alcohol use and smoking habits and periodontal status adjusted for age and sex is shown in Table 3. Individuals who reported hypertension, glaucoma, anxiety and depression exhibited significantly higher odds of having periodontitis (*p* < 0.05). In contrast, patients who reported cancer, alcohol use, kidney problems, asthma, sleep apnea and gastrointestinal disorders were significantly more likely to be associated with periodontal health (*p* < 0.05).

The medication types and the prevalence of consumption in the periodontitis and periodontal health groups are shown in Table 4. Six medication types/groups and forty-five medications were included in the analysis. All six medication types including bisphosphonates, proton pump inhibitors, antidepressants, anticoagulants, statins and ACE inhibitors were significantly associated with periodontitis. When specific medications were analyzed, esomeprazole, omeprazole, apixaban, xarelto, aspirin, clopidogrel, rosuvastatin, pravastatin, atorvastatin, simvastatin, ramipril, enalapril, lisinopril, sertraline and alendronate showed a statistically significantly higher (*p* < 0.05) intake frequency in the periodontitis group relative to the healthy group. 

The multivariable regression analysis for the association between medication intake and periodontal status, adjusted for age and sex, is shown in Table 5. The adjusted odds ratios for the effects of medications between the periodontitis and healthy groups revealed that anticoagulants, statins and ACE inhibitors are significantly (*p* < 0.05) associated with periodontitis. Patients who reported taking anticoagulants, statins and ACE inhibitors demonstrated 3.546 (95% CI: 1.982, 6.343), 2.771 (95% CI: 1.877, 4.090) and 4.847 (95% CI: 2.785, 8.434) times higher odds of having periodontitis, respectively (*p* < 0.001).

## 4. Discussion

Limited information is available regarding the potential association between systemic medication intake and periodontal disease. The present study aimed at evaluating the relationship of medication intake and systemic conditions with periodontitis. Multivariable regression models were performed to explore these possible associations. Based on the data exported from dental charts of adult patients who, seeking dental therapy, had attended the dental clinics of the universities contributing data to the BigMouth network and had a diagnosis of periodontal health or stage III and IV periodontitis, the following findings are reported:

- Older individuals, Hispanic ethnic groups, Black and Hispanic or Latino racial groups and non-White individuals in general were significantly more frequently diagnosed with periodontitis than health.
- After adjusting for the effects of age, sex and missing teeth at baseline, patients with hypertension, glaucoma, anxiety and depression exhibited significantly higher odds of having periodontitis (*p* < 0.05), while patients with cancer, alcohol use, kidney problems, asthma, sleep apnea and gastrointestinal disorders were significantly associated with periodontal health (*p* < 0.005).
- Following the adjustment for age, sex and the number of missing teeth at baseline, anticoagulants, statins and ACE inhibitors were significantly associated with periodontitis.

Diagnoses of moderate-severe and severe periodontitis have been associated with hypertension, while periodontal treatment leads to an improvement in hypertensive and prehypertensive individuals [27,28]. In addition, a systematic review of the literature showed that individuals with tooth loss exhibited a higher risk for hypertension, and hypertensive individuals have a higher risk of tooth loss [29]. Thus, this suggests a bidirectional association between tooth loss and hypertension [30]. In the present investigation, hypertensive patients were more likely associated with periodontitis, which is in agreement with the literature. In addition, glaucoma is a neurodegenerative disease that can be exacerbated by chronic systemic inflammation, which is a characteristic of periodontal disease [31]. It has also been reported that patients with glaucoma exhibit fewer natural teeth and a higher number of oral bacteria [32]. In a large retrospective population-based cohort study (*n* = 194,090) that utilized data from Taiwan’s National Health Insurance Research Database, glaucoma was highly prevalent among periodontitis patients when compared to periodontally healthy individuals, and the authors concluded that patients with periodontitis may increase the risk of glaucoma development compared with individuals without periodontitis [33]. These findings are consistent with the results of the present study. 

In the present investigation, both depression and anxiety were highly associated with periodontitis. This is in agreement with findings reported in the literature. In a multicenter cross-sectional study, both anxiety and depression were highly prevalent among individuals with dental diseases, and a diagnosis of periodontitis was significantly associated with depression [34]. A systematic review of the literature demonstrated that psychological disorders including anxiety and depression were associated with greater tooth loss, while no association was found between psychological conditions and periodontal disease, except for panic disorder [35]. A more recent systematic review and meta-analysis that included 40 studies and evaluated the relationship of periodontal disease with depression and anxiety revealed that both psychological conditions were positively associated with periodontitis [36]. However, the researchers highlighted the high degree of heterogeneity among the publications [36]. Poorer oral hygiene practices, increased tobacco use as well as alcohol consumption, which are characteristics of individuals with mental health disorders, may be significant contributing factors [37]. Also, these associations may be explained by the immunomodulatory effects as well as the increased production of proinflammatory cytokines in individuals with such disorders [37]. Even the interaction of the gut microbiome with the central nervous system and, additionally, the role of the gut microbiome in the oral microbiome may be involved [37]. 

Periodontitis has been associated with a number of systemic conditions. However, the biological connections are still not clearly understood. Several theories have been proposed, including bacteremia, low-grade inflammation and increased myelopoietic activity. Bacteria or activated lymphocytes may disseminate from the oral cavity to distant tissues, leading to inflammatory and functional complications, which may therefore initiate comorbidities [38]. Inflammation in the oral cavity may induce an acute response and metabolic/inflammatory alterations in the liver and bone marrow, which can result in different systemic chronic inflammatory diseases [38]. In addition, patients with severe periodontitis exhibit elevated levels of pro-inflammatory mediators and increased neutrophil counts in the blood, which may explain the periodontal-systemic connection [38]. 

Anti-coagulants and antiplatelet medications including apixaban, plavix, xarelto, brilinta, cilostazol, aspirin, clopidogrel and warfarin were analyzed in the present study. Aspirin was the most frequently used anticoagulant in the study. Individuals who reported taking anticoagulants demonstrated 3.546 (95% CI: 1.982, 6.343) times higher odds of having periodontitis (*p* < 0.001). This may be attributed to the underlying cardiovascular diseases that are associated with chronic inflammation. Limited information is available regarding these medications. Low-dose aspirin has been investigated for its potential anti-inflammatory properties [39]. Low-dose aspirin has shown no association with periodontal status in a nationally representative sample of US adults, while another study demonstrated promising results as a host modulatory treatment when used as an adjunct to periodontal treatment [40,41]. A case-control study that included patients with generalized moderate to severe periodontitis and healthy individuals did not detect any significant associations between periodontal status and anticoagulant treatment [24]. Oral anticoagulants have been associated with an increased risk of osteoporosis due to their effect on bone metabolism and bone mineral density, primarily due to their impact on osteocalcin [42]. This may explain the increased risk of periodontitis in the present population.

In the examined group of statins, atorvastatin, lovastatin, pravastatin, rosuvastatin and simvastatin were included. Individuals who reported statin intake showed 2.771 (95% CI: 1.877, 4.09) times higher odds of having periodontitis (*p* < 0.001). In a similar large longitudinal study that included 169,381 patients prescribed statins from the Korean National Health Insurance Service–Health Screening Cohort database between 2002 and 2015, the presence of chronic periodontitis was significantly more common in patients with long-term statin use (1–3 years, 3–5 years or >5 years) than in those with short-term use (≤1 year) [43]. It was also noted that, among long-term statin users, pre-existing periodontitis was uncommon, while newly occurring periodontitis was common, which reveals the effect of the medication on periodontal status [43]. In addition, the researchers reported a 1.32-fold higher rate of periodontitis occurrence in statin users [43]. It has also been reported in the literature that statin therapy is associated with a slightly increased risk of diabetes development, which may imply a possible dysregulation of the immune response of the host, which is crucial in periodontal stability [44]. Another interpretation is that, due to the anti-inflammatory effect of statins, the immune response may be dampened to protect against periodontal destruction [45]. This may ultimately lead to disruption of the immune homeostasis in periodontal tissues, leading to periodontal tissue breakdown [45]. Furthermore, elevated levels of serum lipids which are commonly found in patients with hypercholesterolemia are associated with periodontal infections [46]. In addition, a retrospective cohort study displayed a significant correlation between systemic statin use and peri-implant bone loss [47]. In contrast, other studies have found that statins may exhibit potential protective effects against tooth loss and may also lead to additional clinical benefits in conjunction with non-surgical periodontal therapy [48,49]. This potential effect has been attributed to their antimicrobial and antibiofilm activity as well as the stimulation of bone activity [50,51]. A dual effect of statins on the periodontium has been described, which is dependent on the inflammatory state of the periodontium [45].

ACE inhibitor use including benazepril, enalapril, lisinopril, quinapril and ramipril with respect to periodontal disease status was also analyzed in the present investigation. ACE inhibitors are commonly used for the treatment of hypertension. In this study, hypertension was found to be significantly associated with the presence of periodontitis, and moreover, the intake of ACE inhibitors was similarly associated with periodontal disease. In agreement with our finding, another study reported that individuals taking ACE inhibitors demonstrated a 3.2-fold higher risk of having sites with a pocket depth ≥ 5 mm and a 2.9-fold higher risk of having sites with clinical attachment loss ≥ 5 mm in comparison with those in the control group [52]. Patients undergoing antihypertensive therapy with ACE inhibitors may have an increased risk of periodontal disease onset as a result of the elevations in kinin activity due to the inactivation of ACE [53] ACE inhibitors may also trigger dendritic cells to produce IL-12 through the activation of B(2) bradykinin receptors, which has been associated with the pathogenesis of periodontal disease [54,55].

Periodontitis is a complex multifactorial chronic inflammatory disease which is a result of the interactions between genetics and environment [18]. In the present study, Hispanic ethnic groups, Black and Hispanic or Latino racial groups and non-White individuals in general were significantly more frequently diagnosed with periodontitis than health. Cultural and genetic factors may contribute to the observed variations in periodontal health among different ethnic and racial groups. In regard to genetics, population-specific risk gene variants have been identified in the literature [56,57]. In addition, periodontitis disparity amongst different racial/ethnic groups has been documented with African Americans and Hispanics demonstrating a higher incidence of periodontitis [58,59].

It is of paramount importance to consider the inherent limitations of this retrospective study. Although it is impossible to demonstrate causality between risk factors and periodontal disease, it may provide evidence for potential risk indicators that can lead to future studies. Future studies may also investigate the impact of socioeconomic factors on medication intake for individuals with periodontitis. The diagnosis of periodontal health and periodontitis was based on the periodontal diagnosis included in the electronic health records of the eligible dental records evaluated. No clinical examination or evaluation of clinical and radiographic data were performed for the purpose of this study. Possible additive, synergistic or antagonistic effects due to drug–drug interactions could not be examined in this investigation. Self-reported medical history and medication intake were utilized in the present investigation to determine their association with periodontal disease. The retrospective study design did not allow for serum collection, which could provide more insights regarding the patients’ medical status. The presence of confounding factors or other biases could not be examined to an extent. Moreover, no information regarding specific dosages or durations of medication intake was available for all of the included patients, and therefore, no statistical analysis was performed. Future studies should further explore the association between medication usage and periodontitis onset and progression. The potential dose- and duration-dependent effects should be examined in the future. Prospective or longitudinal investigations are also needed to include serum-based findings as well as rule out confounding factors. 

A strength of this investigation was the use of data from a large record database (BigMouth) that has in-depth information on patients’ dental and medical histories, derived from a variety of institutions and providers. Pooling datasets from different sources provides greater insight into the types of patients taking specific medications and who may be at risk of periodontitis. In addition, in the published literature, limited data are currently available regarding the effect of systemic medications on periodontal status, and this investigation explores this potential association and provides evidence for possible relationships obtained from a large database of multiple dental schools. Oral health is considered part of general health, and based on the findings of the present investigation, systemic medical conditions and medication intake are associated with periodontal disease. Therefore, both dental clinicians and general practitioners should be aware of these findings to inform their patients about these relationships. The cooperation between medical and dental professionals is crucial. 

## 5. Conclusions

Within the limitations of this retrospective study that utilized the BigMouth dental data repository, there is a possible relationship between systemic medications including anticoagulants, ACE inhibitors and statins as well as systemic medical conditions including hypertension, glaucoma, anxiety and depression and periodontitis. Future studies should further explore the association between medication usage and periodontitis onset and progression in prospective or longitudinal investigations taking into consideration potential confounding factors. The potential dose- and duration-dependent effects should be examined.

## Figures and Tables

**Table 1:** Demographic characteristics of the included population.

Characteristics		Total Population *n* = 1985	Periodontal Health *n* = 886	Periodontitis *n* = 1099	*p*-Value
AGE (mean (SD))		48.34 (17.7)	39.40 (18.3)	55.55 (13.4)	**<0.001**
SEX (%)	FEMALE	1043 (52.5)	462 (52.1)	581 (52.9)	0.783
	MALE	942 (47.5)	424 (47.9)	518 (47.1)	
ETHNICITY (%)	NON-HISPANIC	822 (58.8)	663 (79.6)	159 (28.1)	**<0.001**
	HISPANIC	523 (37.4)	128 (15.4)	395 (69.8)	
	OTHERS	54 (3.9)	42 (5.0)	12 (2.1)	
RACE (%)	WHITE	830 (41.8)	506 (57.1)	324 (29.5)	
	AMERICAN INDIAN	5 (0.3)	2 (0.2)	3 (0.3)	0.353
	ASIAN	206 (10.4)	92 (10.4)	114 (10.4)	**<0.001**
	BLACK	311 (15.7)	95 (10.7)	216 (19.7)	**<0.001**
	HISPANIC OR LATINO	376 (18.9)	2 (0.2)	374 (34.0)	**<0.001**
	OTHERS	154 (7.8)	130 (14.7)	24 (2.2)	**<0.001**
	PACIFIC ISLANDER	2 (0.1)	2 (0.2)	0 (0.0)	0.972
	TWO OR MORE	101 (5.1)	57 (6.4)	44 (4.0)	0.380
RACE White (%)	NON-WHITE	1106 (55.7)	354 (40.0)	752 (68.4)	**<0.001**
	WHITE	879 (44.3)	532 (60.0)	347 (31.6)	
Missing teeth (mean (SD))		1.54 (2.7)	0.46 (1.2)	2.41 (3.2)	**<0.001**

Two sample *t*-tests and Chi-square tests were used for the comparison of continuous and categorical characteristics between healthy and periodontal disease patients. Bold denotes significance.

**Table 2:** Examined self-reported systemic medical conditions as well as alcohol use and smoking habits compared between periodontal health and periodontitis individuals.

Self-Reported Medical Condition, Alcohol Use and Smoking	Periodontal Health *n* = 886	Periodontitis *n* = 1099	*p*-Value
BLOOD HEMOTOLOGICAL DISORDERS (%)	16 (1.8)	27 (2.5)	0.355
CANCER (%)	51 (5.8)	38 (3.5)	**0.016**
ANGINA (%)	3 (0.3)	8 (0.7)	0.364
CONGENITAL HEART DISEASE (%)	5 (0.6)	8 (0.7)	0.783
CORONARY HEART DISEASE (%)	4 (0.5)	25 (2.3)	**0.001**
HEART ATTACK (%)	7 (0.8)	12 (1.1)	0.644
HYPERTENSION (%)	106 (12.0)	447 (40.7)	**<0.001**
IMPLANT DEFIBRILLATOR (%)	6 (0.7)	26 (2.4)	**0.003**
CIGARETTES (%)	59 (6.7)	69 (6.3)	0.783
ALCOHOL USE (%)	454 (51.2)	346 (31.5)	**<0.001**
MARIJUANA USE (%)	21 (2.4)	45 (4.1)	**0.043**
DIABETES (%)	55 (6.2)	127 (11.6)	**<0.001**
THYROID PROBLEMS (%)	52 (5.9)	71 (6.5)	0.640
GLAUCOMA (%)	9 (1.0)	167 (15.2)	**<0.001**
HIV (%)	1 (0.1)	4 (0.4)	0.389
SEXUALLY TRANSMITTED DISEASES (%)	3 (0.3)	14 (1.3)	**0.027**
DIALYSIS (%)	2 (0.2)	5 (0.5)	0.471
KIDNEY (%)	46 (5.2)	55 (5.0)	0.918
RENAL FAILURE OR INSUFFICIENCY (%)	8 (0.9)	19 (1.7)	0.123
ARTHRITIS (%)	70 (7.9)	203 (18.5)	**<0.001**
OSTEOPOROSIS (%)	10 (1.1)	67 (6.1)	**<0.001**
ANXIETY (%)	20 (2.3)	72 (6.6)	**<0.001**
DEPRESSION (%)	16 (1.8)	80 (7.3)	**<0.001**
PARKINSON’S DISEASE (%)	1 (0.1)	4 (0.4)	0.389
MULTIPLE SCLEROSIS (%)	2 (0.2)	1 (0.1)	0.589
SEIZURE OR EPILEPSY (%)	9 (1.0)	11 (1.0)	>0.99
STROKE (%)	4 (0.5)	13 (1.2)	0.090
ORGAN TRANSPLANT (%)	5 (0.6)	6 (0.5)	>0.99
ASTHMA (%)	54 (6.1)	3 (0.3)	**<0.001**
CHRONIC BRONCHITIS OR EMPHYSEMA (%)	7 (0.8)	9 ( 0.8)	>0.99
SLEEP APNEA (%)	20 (2.3)	3 (0.3)	**<0.001**
CROHN’S DISEASE (%)	4 (0.5)	7 (0.6)	0.764
GASTROINTESTINAL DISORDERS (%)	50 (5.6)	5 (0.5)	**<0.001**
HEPATITIS (%)	16 (1.8)	55 (5.0)	**<0.001**
ANEMIA (%)	16 (1.8)	0 (0.0)	**<0.001**
BLEEDING DISORDERS (%)	4 (0.5)	0 (0.0)	**0.040**
LEUKEMIA (%)	3 (0.3)	0 (0.0)	0.089
CARDIOVASCULAR DISEASES (%)	170 (19.2)	0 (0.0)	**<0.001**
HISTORY OF ENDOCARDITIS (%)	1 (0.1)	0 (0.0)	0.446
COCAINE USE (%)	1 (0.1)	0 (0.0)	0.446
METHAMPHETAMINE USE (%)	1 (0.1)	0 (0.0)	0.446
ANOREXIA (%)	6 (0.7)	0 (0.0)	**0.008**
BULIMIA (%)	5 (0.6)	0 (0.0)	**0.018**
BRONCHITIS (%)	50 (5.6)	0 (0.0)	**<0.001**
RESPIRATORY OR LUNG PROBLEM (%)	49 (5.5)	0 (0.0)	**<0.001**
SINUSITIS (%)	83 (9.4)	0 (0.0)	**<0.001**
TUBERCULOSIS (%)	25 (2.8)	0 (0.0)	**<0.001**
CIRRHOSIS OR CHRONIC HEPATITIS (%)	18 (2.0)	0 (0.0)	**<0.001**
RHEUMATIC FEVER (%)	0 (0.0)	8 (0.7)	**0.010**
ANDRENAL GLAND DISORDER (%)	0 (0.0)	2 (0.2)	0.506
LUPUS (%)	0 (0.0)	2 (0.2)	0.506
DEMENTIA (%)	0 (0.0)	4 (0.4)	0.133

Bold denotes significance. Univariate associations between systemic medical conditions and periodontal disease status were assessed using a logistic regression model.

**Table 3:** Multivariable regression analysis for the association between systemic medical conditions, alcohol use, smoking habits and periodontal status adjusted for age and sex.

Self-Reported Medical Condition, Alcohol Use and Smoking	Adjusted OR (95% CI)	*p*-Value
BLOOD HEMOTOLOGICAL DISORDERS	0.647 (0.307, 1.364)	0.998
CANCER	0.161 (0.094, 0.271)	**<0.001**
ANGINA	1.484 (0.343, 6.426)	0.998
CONGENITAL HEART DISEASE	0.412 (0.106, 1.607)	0.998
CORONARY HEART DISEASE	1.161 (0.376, 3.591)	0.998
HEART ATTACK	0.385 (0.137, 1.082)	0.998
HYPERTENSION	2.035 (1.536, 2.682)	**<0.001**
IMPLANT DEFIBRILLATOR	1.181 (0.421, 3.317)	0.998
CIGARETTES	0.537 (0.351, 0.822)	0.097
ALCOHOL USE	0.459 (0.367, 0.574)	**<0.001**
MARIJUANA USE	2.251 (1.201, 4.221)	0.262
DIABETES	0.786 (0.535, 1.155)	0.998
THYROID PROBLEMS	0.639 (0.412, 0.997)	0.998
GLAUCOMA	8.368 (4.102, 17.069)	**<0.001**
HIV	2.388 (0.241, 23.700)	0.998
SEXUALLY TRANSMITTED DISEASES	2.498 (0.606, 10.296)	0.998
DIALYSIS	0.840 (0.124, 5.686)	0.998
KIDNEY	0.401 (0.249, 0.646)	**0.004**
RENAL FAILURE OR INSUFFICIENCY	0.863 (0.337, 2.215)	0.998
ARTHRITIS	1.088 (0.774, 1.529)	0.998
OSTEOPOROSIS	2.327 (1.139, 4.755)	0.452
ANXIETY	2.651 (1.499, 4.688)	**0.020**
DEPRESSION	3.864 (2.102, 7.103)	**<0.001**
PARKINSON’S DISEASE	0.997 (0.101, 9.977)	0.998
MULTIPLE SCLEROSIS	0.402 (0.030, 5.332)	0.998
SEIZURE OR EPILEPSY	0.803 (0.271, 2.393)	0.998
STROKE	1.011 (0.301, 3.396)	0.998
ORGAN TRANSPLANT	0.372 (0.104, 1.332)	0.998
ASTHMA	0.019 (0.005, 0.081)	**<0.001**
CHRONIC BRONCHITIS OR EMPHYSEMA	0.589 (0.185, 1.875)	0.998
SLEEP APNEA	0.034 (0.008, 0.138)	**<0.001**
CROHN’S DISEASE	1.841 (0.432, 7.856)	0.998
GASTROINTESTINAL DISORDERS	0.062 (0.022, 0.175)	**<0.001**
HEPATITIS	1.389 (0.743, 2.612)	0.998

Bold denotes significance. Multivariable regression models were explored, adjusting for the effects of age, sex and missing teeth at baseline. *p*-values were corrected for multiple comparisons using the Hochberg method.

**Table 4:** Medication types and prevalence of consumption in the periodontitis and periodontal health groups.

Medications	Periodontal Health *n* = 886	Periodontitis *n* = 1099	*p*-Value
BIPHOSPHONATES (%)	6 (0.7)	22 (2.0)	**0.022**
PROTON PUMP INHIBITORS (%)	20 (2.3)	83 (7.6)	**<0.001**
ANTIDEPRESSANTS (%)	56 (6.3)	105 (9.6)	**0.011**
ANTICOAGULANTS (%)	15 (1.7)	140 (12.7)	**<0.001**
STATINS (%)	38 (4.3)	239 (21.7)	**<0.001**
ACE INHIBITORS (%)	16 (1.8)	154 (14.0)	**<0.001**
PANTOPRAZOLE (%)	1 (0.1)	25 (2.3)	**<0.001**
LANSOPRAZOLE (%)	3 (0.3)	1 (0.1)	0.330
ESOMEPRAZOLE (%)	3 (0.3)	14 (1.3)	**0.027**
DEXILANT (%)	1 (0.1)	1 (0.1)	>0.99
OMEPRAZOLE (%)	16 (1.8)	43 (3.9)	**0.007**
APIXABAN (%)	5 (0.6)	19 (1.7)	**0.022**
PLAVIX (%)	3 (0.3)	10 (0.9)	0.162
XARELTO (%)	1 (0.1)	8 (0.7)	**0.048**
BRILINTA (%)	1 (0.1)	1 (0.1)	>0.99
CILOSTAZOL (%)	0 (0.0)	2 (0.2)	0.506
ASPIRIN (%)	4 (0.5)	102 (9.3)	**<0.001**
CLOPIDOGREL (%)	2 (0.2)	14 (1.3)	**0.010**
WARFARIN (%)	2 (0.2)	8 (0.7)	0.200
ROSUVASTATIN (%)	5 (0.6)	30 (2.7)	**<0.001**
PRAVASTATIN (%)	5 (0.6)	26 (2.4)	**0.002**
LOVASTATIN (%)	4 (0.5)	11 (1.0)	0.197
PITAVASTATIN (%)	0 (0.0)	1 (0.1)	>0.99
FLUVASTATIN (%)	0 (0.0)	1 (0.1)	>0.99
ATORVASTATIN (%)	17 (1.9)	120 (10.9)	**<0.001**
SIMVASTATIN (%)	7 (0.8)	61 (5.6)	**<0.001**
BENAZEPRIL (%)	1 (0.1)	3 (0.3)	0.633
QUINAPRIL (%)	2 (0.2)	4 (0.4)	0.698
RAMIPRIL (%)	0 (0.0)	6 (0.5)	**0.036**
ENALAPRIL (%)	1 (0.1)	9 (0.8)	**0.050**
LISINOPRIL (%)	12 (1.4)	132 (12.0)	**<0.001**
FLUOXETINE (%)	4 (0.5)	8 (0.7)	0.565
PAROXETINE (%)	3 (0.3)	3 (0.3)	>0.99
SERTRALINE (%)	3 (0.3)	18 (1.6)	**0.007**
CITALOPRAM (%)	14 (1.6)	32 (2.9)	0.052
DULOXETINE (%)	5 (0.6)	11 (1.0)	0.322
VENLAFAXINE (%)	7 (0.8)	4 (0.4)	0.235
DESVENLAFAXINE (%)	2 (0.2)	1 (0.1)	0.589
TRAZODONE (%)	4 (0.5)	13 (1.2)	0.090
MIRTAZAPINE (%)	1 (0.1)	3 (0.3)	0.633
BUPROPION (%)	8 (0.9)	21 (1.9)	0.089
NORTRIPTYLINE (%)	1 (0.1)	2 (0.2)	>0.99
DOXEPIN (%)	0 (0.0)	1 (0.1)	>0.99
ARIPIPRAZOLE (%)	1 (0.1)	3 (0.3)	0.633
QUETIAPINE (%)	3 (0.3)	5 (0.5)	0.738
LITHIUM (%)	0 (0.0)	1 (0.1)	>0.99
AMITRYPTILINE (%)	9 (1.0)	6 (0.5)	0.299
ZOLEDRONIC ACID (%)	2 (0.2)	0 (0.0)	0.199
IBANDRONATE (%)	0 (0.0)	1 (0.1)	>0.99
ALENDRONATE (%)	4 (0.5)	20 (1.8)	**0.006**
RISEDRONATE (%)	0 (0.0)	1 (0.1)	>0.99

Bold denotes significance. Univariate associations between medications and periodontal disease status were assessed using a logistic regression model.

**Table 5:** Multivariable analysis for the association between medication intake and periodontal status (healthy vs. periodontitis), adjusted for age and sex.

Medications	Adjusted OR (95% CI)	*p*-Value
BIPHOSPHONATES	1.179 (0.442, 3.141)	0.864
PROTON PUMP INHIBITORS	1.491 (0.853, 2.607)	0.483
ANTIDEPRESSANTS	0.966 (0.652, 1.431)	0.864
ANTICOAGULANTS	3.546 (1.982, 6.343)	**<0.001**
STATINS	2.771 (1.877, 4.090)	**<0.001**
ACE INHIBITORS	4.847 (2.785, 8.434)	**<0.001**

Bold denotes significance. Multivariable regression models were explored, adjusting for the effects of age, sex and missing teeth at baseline. *P*-values were corrected for multiple comparisons using the Hochberg method.

## Data Availability

The datasets used and/or analyzed during the current study are available from the corresponding author on reasonable request.
